# Disrupted local functional connectivity in schizophrenia: An updated and extended meta-analysis

**DOI:** 10.1038/s41537-022-00311-2

**Published:** 2022-11-08

**Authors:** Mengjing Cai, Rui Wang, Mengge Liu, Xiaotong Du, Kaizhong Xue, Yuan Ji, Zirui Wang, Yijing Zhang, Lining Guo, Wen Qin, Wenshuang Zhu, Jilian Fu, Feng Liu

**Affiliations:** 1 Department of Radiology and Tianjin Key Laboratory of Functional Imaging, Tianjin Medical University General Hospital, Tianjin, 300052 China; 2 School of Medical Imaging, Tianjin Medical University, Tianjin, 300070 China; 3 Department of Radiology, Shanghai Ninth People’s Hospital, Shanghai Jiao Tong University School of Medicine, Shanghai, 200011 China

**Keywords:** Schizophrenia, Schizophrenia

## Abstract

Neuroimaging studies have shown that schizophrenia is associated with disruption of resting-state local functional connectivity. However, these findings vary considerably, which hampers our understanding of the underlying pathophysiological mechanisms of schizophrenia. Here, we performed an updated and extended meta-analysis to identify the most consistent changes of local functional connectivity measured by regional homogeneity (ReHo) in schizophrenia. Specifically, a systematic search of ReHo studies in patients with schizophrenia in PubMed, Embase, and Web of Science identified 18 studies (20 datasets), including 652 patients and 596 healthy controls. In addition, we included three whole-brain statistical maps of ReHo differences calculated based on independent datasets (163 patients and 194 controls). A voxel-wise meta-analysis was then conducted to investigate ReHo alterations and their relationship with clinical characteristics using the newly developed seed-based *d* mapping with permutation of subject images (SDM-PSI) meta-analytic approach. Compared with healthy controls, patients with schizophrenia showed significantly higher ReHo in the bilateral medial superior frontal gyrus, while lower ReHo in the bilateral postcentral gyrus, right precentral gyrus, and right middle occipital gyrus. The following sensitivity analyses including jackknife analysis, subgroup analysis, heterogeneity test, and publication bias test demonstrated that our results were robust and highly reliable. Meta-regression analysis revealed that illness duration was negatively correlated with ReHo abnormalities in the right precentral/postcentral gyrus. This comprehensive meta-analysis not only identified consistent and reliably aberrant local functional connectivity in schizophrenia but also helped to further deepen our understanding of its pathophysiology.

## Introduction

Schizophrenia is a complex and chronic psychiatric disorder with a lifetime prevalence of approximately 1%, and is characterized by a combination of psychotic symptoms such as positive symptoms (delusions and hallucinations), negative symptoms (affective flattening, amotivation, and social withdrawal), and cognitive dysfunctions^1^. The associated symptoms and cognitive changes have been attributed to abnormal brain connectivity in schizophrenia^2^. Although substantial efforts have been made over the past few decades, the exact pathophysiological mechanisms of schizophrenia remain largely unknown.

Recent advances in neuroimaging techniques have made it possible to elucidate the neurobiological basis of schizophrenia. Among them, resting-state functional magnetic resonance imaging (fMRI) is increasingly used to investigate brain functional alterations in clinical studies, which requires minimal patient compliance and avoids potential constraints of task-dependent paradigms^3^. In previous resting-state fMRI studies, functional connectivity (FC) via seed-based analysis or independent component analysis (ICA) was often used to investigate brain functional dysconnectivity in schizophrenia^4,5^. These methods mainly focus on the temporal coincidence between spatially distinct brain regions or within a certain resting-state network, rather than local connectivity between spatially adjacent regions. As a complementary approach, regional homogeneity (ReHo), defined by the temporal coherence or synchronization of the time series within a local area, could be used to measure local FC^6,7^. ReHo is a whole-brain level metric with high test-retest reliability, and it has been widely used to measure local FC under neurocognitive and neuropsychiatric conditions^8,9^. To date, several studies have employed ReHo to investigate aberrant local FC in schizophrenia, but the findings are inconsistent and even conflicting. For example, some studies only found decreased ReHo^10,11^, while others only observed increased ReHo in patients with schizophrenia^12,13^. Additionally, in separate studies, ReHo was also found to decrease or increase in different brain regions in patients with schizophrenia^14,15^. This discrepancy may be attributed to between-study variations in demographic and clinical characteristics of patients, and approaches of image data acquisition and analysis; the relatively small sample size in these studies may also account for the distinct findings.

Neuroimaging meta-analysis has emerged as a powerful method to integrate findings of different studies and can effectively address problems related to individual studies^16,17^. To our knowledge, several studies have conducted coordinate-based meta-analyses (CBMAs) to investigate ReHo changes in schizophrenia^18–21^, but the results are still inconsistent. Among these studies, two studies used the activation likelihood estimation (ALE) method in meta-analyses^18,19^, which may lead to opposite directions in the same voxel erroneously because the coordinates of increased and decreased results are separately calculated in ALE^22^. Another two employed the anisotropic effect-size version of seed-based *d* mapping (AES-SDM) method^20,21^, which has the advantage over ALE for combining both positive and negative alterations to avoid contradictory findings in the same voxel^22^. However, AES-SDM progressively estimates the effect sizes of voxels close to the reported peaks, which may introduce inevitable bias^23^. In addition, the aforementioned CBMAs included a relatively small number of studies, and did not include several newly published ReHo studies in schizophrenia^24–27^. Furthermore, they involved a dramatic loss of information due to the use of only a list of local maxima coordinates. Instead, image-based meta-analysis (IBMA) uses original whole-brain statistical parametric maps, substantially increases the statistical power, and is superior to CBMA^28^.

In the present study, we conducted an updated and extended whole-brain voxel-wise meta-analysis to summarize available neuroimaging findings of ReHo changes in schizophrenia via the seed-based *d* mapping with permutation of subject images (SDM-PSI) software. Specifically, this study was updated by integrating newly published ReHo studies in schizophrenia, and extended by including three independent neuroimaging datasets of schizophrenia. Compared with AES-SDM, SDM-PSI has several novel features such as using standard voxel-wise tests, less biased estimation of the population effect size, and multiple imputation of study images to avoid biases related to single imputation^23^. Therefore, the purpose of the current study was mainly twofold: we first sought to identify consistent and reliable ReHo changes in schizophrenia; second, we aimed to explore whether ReHo alterations were associated with clinical variables in schizophrenia. To this aim, we first comprehensively searched published ReHo studies in schizophrenia and collected peak coordinate information. Then, three independent resting-state fMRI datasets of schizophrenia were downloaded from the publicly available SchizConnect database (http://schizconnect.org/), between-group ReHo comparisons were performed, and whole-brain statistical maps were obtained. Subsequently, a combined coordinate- and image-based meta-analysis was conducted to investigate ReHo changes. Finally, a series of subgroup meta-analyses and meta-regression analyses were carried out to explore the potential impacts of demographic, methodological, or clinical characteristics on ReHo alterations.

## Materials and methods

### Literature search and selection

**Fig. 1:**
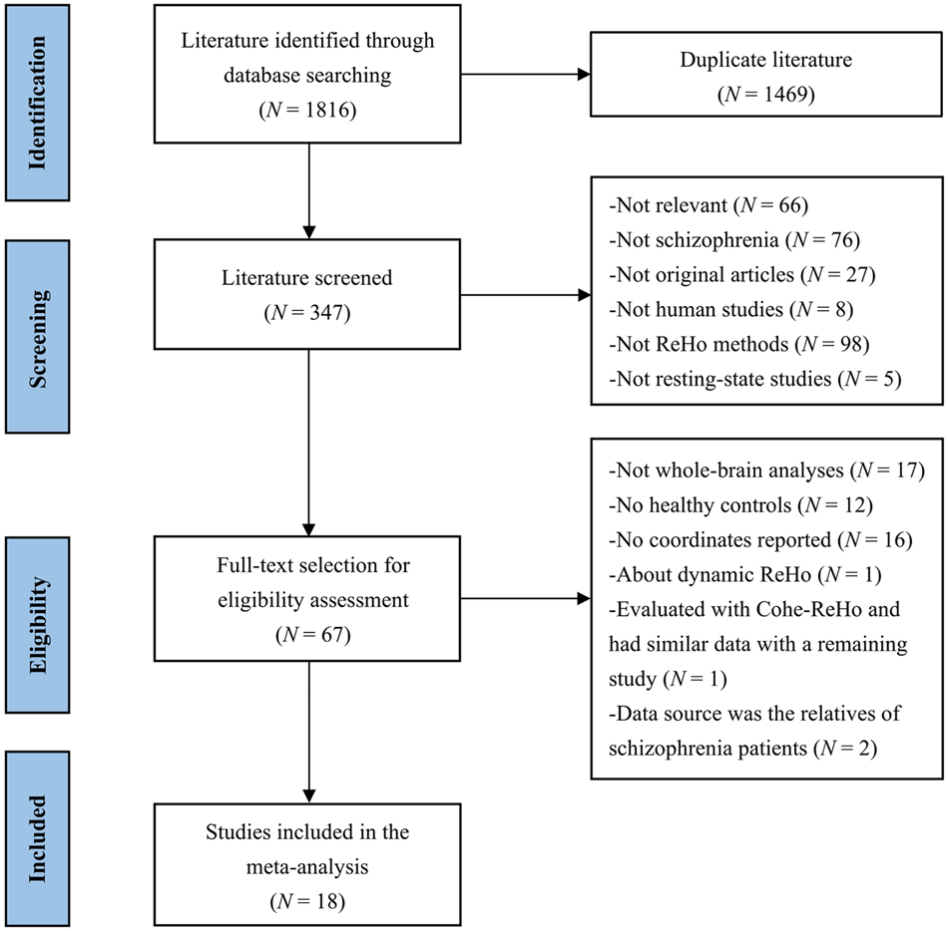
The flowchart of study selection according to the Preferred Reporting Items for Systematic Reviews and Meta-Analyses (PRISMA) guidelines. Abbreviations: N number; Cohe-ReHo coherence-based regional homogeneity.

A comprehensive and exhaustive computerized search of relevant studies published in PubMed, Embase, and Web of Science databases before March 2022 was conducted according to the following key terms: (“schizophrenia” or “schizophrenics” or “schizophrenic disorder”) and (“ReHo” or “regional homogeneity” or “local consistency” or “local coherence” or “local connectivity”) and (“fMRI” or “functional magnetic resonance imaging” or “resting-state”). The reference lists of eligible studies and relevant previous reviews or meta-articles were also manually searched. The studies were included if they met all the following criteria: (1) the patients met the diagnostic criteria for schizophrenia; (2) the studies were original research and peer-reviewed published in English-language journals; (3) the ReHo comparison was conducted between patients with schizophrenia and healthy controls; (4) the whole-brain analysis was conducted; (5) three-dimensional peak coordinates in Talairach or Montreal Neurological Institute (MNI) space were provided, or null findings were reported. The exclusion criteria were as follows: (1) the studies only reported results obtained from the region of interest analysis, partial coverage analysis, or small volume correction; (2) inconsistent thresholds were used in different regions; (3) the number of subjects was less than 10 in either patient or control groups. If the recruited data were from the same resources, studies with the largest sample sizes were preferred. Furthermore, the corresponding author was contacted for details if the information provided was not comprehensive. Our meta-analysis was conducted based on the Preferred Reporting Items for Systematic Reviews and Meta-Analyses (PRISMA) guidelines, and the detailed study selection procedures are summarized in Fig. 1.

Two authors independently searched and checked the literature, and the following information from each included study was extracted, including demographic (e.g., sample size, mean age, and gender) and clinical (e.g., medication status and illness duration) characteristics, data acquisition and processing methods (e.g., MRI scanner, slice thickness, and smooth kernel size), peak coordinates and statistics (e.g., *t*-values).

### Quality assessment of selected studies

The quality of included studies was assessed with a 10-point checklist which is in line with previous studies, mainly including the demographic and clinical characteristics of the patients, analysis methods, and the quality of results and conclusion^29,30^. The score of each item was given as 0, 0.5, and 1 according to whether the criteria were not, partially, or fully met, respectively. It is noteworthy that the result of the checklist did not represent the quality of the studies, but just referred to the degree of satisfying the included criteria in our study. The detailed checklist and the scores for each study are shown in Tables S1 and S2.

### Neuroimaging data processing and ReHo comparison

Three publicly available resting-state fMRI datasets of schizophrenia were downloaded from SchizConnect^31^, including the BrainGluSchi^32^, COBRE^33^, and NMorphCH datasets^34^. The detailed information including participant inclusion and exclusion criteria, and imaging acquisition details of the public datasets are available on the SchizConnect website (http://schizconnect.org/), and only the baseline data in the NMorphCH dataset were included in this study. All experimental procedures were approved by the local Ethics Committee and informed written consent was provided by each participant.

The image quality was checked by two radiologists. We checked head motion with the realignment procedure in data preprocessing (see immediately below), and the participants with head movements exceeding 3.0 mm of translation or 3.0 degrees of rotation in any direction were excluded. After that, 58 patients and 77 controls in the BrainGluSchi dataset, 71 patients and 84 controls in the COBRE dataset, and 34 patients and 33 controls in the NMorphCH dataset were included. The resting-state fMRI was processed in each dataset with the following steps: (1) time points of the first 20 s were removed to allow the signal to reach equilibrium; (2) slice timing and realignment were performed for the remaining fMRI time series to correct within-scan acquisition time differences and head motion, respectively; (3) the corrected fMRI data were normalized to standard MNI space based on the parameters derived from new segmentation and DARTEL algorithm of the structural MRI data; (4) nuisance covariates including linear trend, global signal, white matter signal, cerebrospinal fluid signal, Friston-24 head motion parameters^35^, and volumes with spike head motion measured with framewise displacement (FD > 0.5)^36^ were regressed out; (5) temporal bandpass filtering (0.01–0.1 Hz) was applied; (6) ReHo was calculated using Kendall’s coefficient of concordance (KCC) according to previous studies^37,38^, and the ReHo map was standardized using *z*-score standardization; (7) the ReHo map was smoothed with a Gaussian kernel of 8 mm full width at half-maximum (FWHM). After data processing, a between-group ReHo comparison was performed using the general linear model with age, gender, and mean FD as covariates, and the raw statistical map (i.e., *t*-map) was obtained in each dataset. Then we used subject-based nonparametric permutation tests (1000 times) with the threshold-free cluster enhancement (TFCE) method to control the family-wise error (FWE) rate, and the significance level was set at FWE *p* < 0.05.

### Voxel-wise meta-analysis

**Fig. 2:**
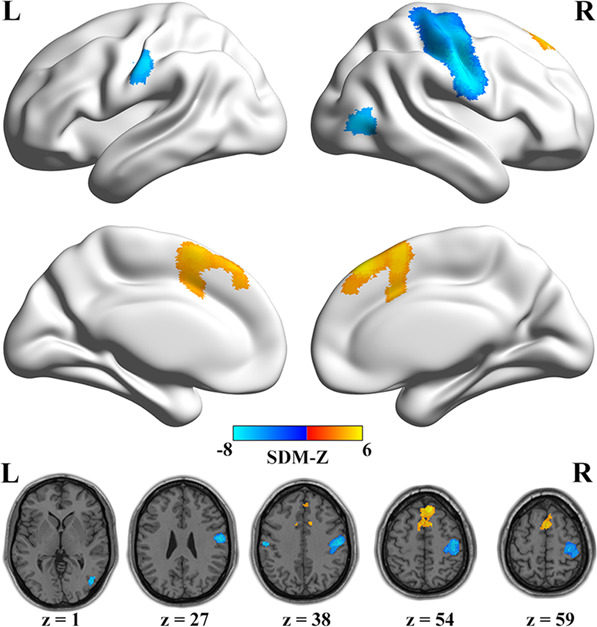
Meta-analysis results of significant ReHo changes in schizophrenia. The colorbar represents SDM-Z value with warm and cold color representing increased and decreased ReHo in patients with schizophrenia, respectively. Abbreviations: L left; R right; ReHo regional homogeneity; SDM seed-based *d* mapping.

To investigate the ReHo alterations in schizophrenia, a voxel-wise meta-analysis was performed by combining peak coordinates and original *t*-maps with the SDM-PSI software (version 6.21, https://www.sdmproject.com/). Briefly, the procedures mainly included (1) multiple imputation of study images (50 times, only for the included coordinate-based studies in Fig. 1), (2) imputation of subject images, (3) group analyses of the subject images for each study and imputation, (4) meta-analyses of the study images using the random-effects model for each imputation, and (5) combination of the meta-analysis results using Rubin’s rules^23^. The statistical significance level was set at FWE-TFCE *p* < 0.05.

### Sensitivity analyses

To test the reliability and reproducibility of the results, we performed four sensitivity analyses. First, a voxel-based jackknife sensitivity analysis was performed by iteratively repeating the same voxel-wise meta-analysis, with a different dataset excluded each time. The results were regarded as reliable if a certain brain region of significant differences remains significant in most repeats. At present, we found no clear criteria on the selection of threshold for jackknife sensitivity analysis used to determine whether the main results are robust^39–41^, and therefore we used the threshold of 80%. Second, the subgroup analysis including only homogenous studies was performed to examine the potential factors influencing ReHo differences when the number of studies was sufficient^42^. Specifically, we conducted subgroup meta-analyses of (1) adult participants, (2) drug-naive/free patients, (3) medicated patients, (4) scanned using a Siemens 3.0T MRI scanner, (5) scanned using repetition time (TR) = 2000 ms, and (6) smoothed with 4 mm FWHM kernel. Given the insufficient datasets, we did not perform additional subgroup analyses. Third, between-study heterogeneity was examined to explore whether our findings were heterogeneous based on Cochran’s *Q* test (approximately follows a chi-square distribution with *N* - 1 degrees of freedom, *N* is the number of studies), and the fraction of variance due to heterogeneity was estimated with *I*^2^ statistics^43^. The *p*_Cochran’s *Q*_ < 0.05 indicates significant between-study heterogeneity; the value of *I*^2^ index ranges from 0 to 100%, with the percentages of around 25, 50, and 75% representing low, medium, and high heterogeneity, respectively^44^. Finally, funnel plot and Egger’s test were used to visually and quantitatively examine the possibility of publication bias for the significant results.

### Meta-regression analysis

To find potential effects of relevant clinical and demographic variables, including illness duration, Positive and Negative Syndrome Scale (PANSS) scores, mean age, and female-to-male ratio, on ReHo differences, we performed meta-regression analyses. Specifically, the mean effect size (i.e., Hedges’ *g*) of each significant cluster was extracted from each study and each imputed dataset, then linear regression analyses were conducted across studies in each imputed dataset between effect size and these study-level continuous variables, and the results from all imputed datasets were pooled using Rubin’s rules^45^. The significance level was set at uncorrected *p* < 0.05.

## Results

### Included studies

As shown in Fig. 1, our search strategy initially identified 1816 studies, of which 18 studies were eligible for meta-analysis including a total of 652 patients with schizophrenia and 596 healthy controls^10–15,24–27,46–53^. Among these studies, three studies were longitudinal designs, and we only included the results of between-group comparison using baseline data^13,46,51^. Additionally, two studies enrolled subgroups of patients with schizophrenia: one study included subgroups of patients with and without auditory verbal hallucinations^47^, and the other contained subgroups of treatment-resistant and non-treatment-resistant patients^49^. Therefore, we treated these studies as comprising two unique and independent datasets. Finally, a total of 20 datasets along with 133 peak coordinates were included in the meta-analysis, and the distribution of these coordinates is shown in Fig. S1.

Sample size weighted *t*-tests revealed that patient group did not significantly differ in age (*p* = 0.420) and gender ratio (female-to-male ratio, *p* = 0.500) compared with control group. The demographic and clinical characteristics of the included studies in the meta-analysis are summarized in Table 1, and a summary of the imaging and technical parameters is shown in Table 2.

**Table 1:** Demographic and clinical characteristics of the studies included in the meta-analysis.

Study	Sample size (female)		Mean age (y)		Education (y)		Age at onset (y)	Duration (m)	PANSS			Medication (%)	First episode
	SZ	HC	SZ	HC	SZ	HC			Total	Positive	Negative		
Bai et al. (2016)	17 (3)	17 (3)	26.00	28.71	10.59	13.65	NA	40.29	82.06	15.29	24.88	Drug free	Partial
Cui et al. (2016)^a^	17 (7)	19 (9)	21.24	23.79	13.71	14.74	NA	6.51	106.24	31.12	25.53	Drug naive	Yes
Cui et al. (2016)^a^	15 (7)	19 (9)	22.53	23.79	13.40	14.74	NA	10.20	88.07	17.93	22.73	Drug naive	Yes
Gao et al. (2015)	14 (5)	14 (5)	33.20	34.90	11.70	11.30	NA	9.20	74.10	16.40	22.60	100	No
Gao et al. (2018)^a^	17 (7)	29 (13)	31.24	32.73	12.24	14.28	17.24	14.00	97.76	27.53	21.05	100	No
Gao et al. (2018)^a^	17 (8)	29 (13)	36.82	32.73	13.76	14.28	29.18	7.88	37.29	9.53	8.41	100	No
Gao et al. (2020)	57 (37)	50 (27)	31.63	28.38	12.86	15.64	NA	2.52	91.84	26.39	20.68	Drug naive	No
Gou et al. (2018)	28 (12)	21 (7)	23.90	28.80	12.90	12.90	NA	15.10	85.70	17.80	21.00	100	No
Hu et al. (2016)	42 (15)	38 (13)	24.86	24.76	10.48	11.05	NA	8.38	91.90	25.60	18.17	Drug naive	Yes
Jin et al. (2021)	23 (12)	24 (12)	31.74	30.92	12.57	14.04	NA	NA	NA	NA	NA	Drug naive	Yes
Liu et al. (2006)	18 (9)	18 (9)	23.67	24.44	14.11	15.28	NA	26.83	80.39	NA	NA	Drug free	No
Liu et al. (2016)	27 (12)	27 (9)	25.44	27.44	12.30	12.96	NA	18.32	85.78	21.56	23.15	44	No
Lyu et al. (2021)	32 (17)	27 (17)	16.75	16.40	10.19	10.11	NA	9.19	79.44	22.75	16.97	Drug naive	Yes
Shan et al. (2021)	39 (12)	20 (6)	24.36	25.70	11.13	12.75	NA	5.00	103.90	23.82	27.28	Drug free	No
Wang et al. (2017)	48 (27)	31 (17)	15.79	15.42	8.88	8.44	NA	5.35	75.10	21.50	17.92	Drug naive	No
Yan et al. (2020)	69 (19)	74 (29)	24.22	26.27	13.23	14.69	NA	13.74	84.19	24.42	17.58	Drug naive	Yes
Yang et al. (2021)	37 (28)	39 (30)	39.70	40.94	10.35	9.56	22.70	204.00	76.00	13.60	23.90	100	No
Yu et al. (2013)	69 (36)	62 (NA)	31.70	29.90	14.20	15.30	NA	7.10	52.90	12.10	13.40	100	No
Yu et al. (2021)	22 (11)	60 (22)	33.41	32.87	10.77	14.02	NA	15.48	93.50	27.18	19.82	Drug naive	No
Zhao et al. (2019)	44 (13)	26 (9)	23.70	22.60	12.80	13.90	NA	12.00	102.00	15.30	24.70	Drug naive	Yes
BrainGluSchi	58 (6)	77 (27)	35.26	38.86	NA	NA	20.17	181.08	61.02^b^ (*n* = 53)	16.36^b^ (*n* = 56)	15.67^b^ (*n* = 55)	90	NA
COBRE	71 (13)	84 (23)	36.62	38.87	NA	NA	21.12^b^ (*n* = 69)	182.76^b^ (*n* = 69)	59.23^b^ (*n* = 69)	15.15	15.31^b^ (*n* = 70)	97	NA
NMorphCH	34 (7)	33 (16)	33.94	29.33	13.00^b^ (*n* = 26)	16.23^b^ (*n* = 22)	18.75^b^ (*n* = 25)	188.76^b^ (*n* = 25)	NA	NA	NA	NA	NA

Note: ^a^The study consisted of two subgroups, and we treated it as two separate datasets. ^b^The information of some participants was not available, and the number of samples with which were provided in parentheses.

Abbreviations: *HC* healthy control; *m* month; *NA* not available; *PANSS* Positive and Negative Syndrome Scale; *SZ* schizophrenia; *y* year.

**Table 2:** Technique details of the studies included in the meta-analysis.

Study	MRI scanner	Head coil	TR (ms)	TE (ms)	ST (mm)	FWHM (mm)	Threshold	Coordinates
Bai et al. (2016)	GE (1.5T)	NA	2000	40	5	4	*p* < 0.05 (Corrected)	15
Cui et al. (2016)^a^	Siemens (3.0T)	8-channel phased array	2000	30	4	NA	*p* < 0.01 (Corrected)	3
Cui et al. (2016)^a^	Siemens (3.0T)	8-channel phased array	2000	30	4	NA	*p* < 0.01 (Corrected)	2
Gao et al. (2015)	Siemens (1.5T)	NA	2000	40	5	6	*p* < 0.05 (Corrected)	2
Gao et al. (2018)^a^	Siemens (3.0T)	Birdcage	2000	30	4	4	*p* < 0.05 (Corrected)	10
Gao et al. (2018)^a^	Siemens (3.0T)	Birdcage	2000	30	4	4	*p* < 0.05 (Corrected)	6
Gao et al. (2020)	Siemens (3.0T)	Birdcage	2000	30	4	4	*p* < 0.05 (Corrected)	3
Gou et al. (2018)	GE (1.5T)	NA	2000	40	5	4	*p* < 0.001 (Corrected)	2
Hu et al. (2016)	Siemens (3.0T)	NA	2000	30	3	8	*p* < 0.005 (Uncorrected)	2
Jin et al. (2021)	Philips (3.0T)	Soft head coil	2000	35	5	6	*p* < 0.05 (Corrected)	2
Liu et al. (2006)	GE (1.5T)	Quadrature birdcage	2000	40	5	4	*p* < 0.05 (Corrected)	28
Liu et al. (2016)	GE (1.5T)	NA	2000	40	5	8	*p* < 0.05 (Corrected)	4
Lyu et al. (2021)	Philips (3.0T)	NA	2000	35	5	6	*p* < 0.05 (Corrected)	1
Shan et al. (2021)	Siemens (3.0T)	NA	2000	30	4	4	*p* < 0.05 (Corrected)	13
Wang et al. (2017)	Siemens (3.0T)	NA	2000	30	4	4	*p* < 0.005 (Corrected)	5
Yan et al. (2020)	Siemens (3.0T)	Standard head coil	2500	30	3.5	4	*p* < 0.05 (Corrected)	3
Yang et al. (2021)	Philips (3.0T)	16-channel phased-array	2200	35	3	4	*p* < 0.05 (Corrected)	10
Yu et al. (2013)	Siemens (3.0T)	32-channel	2000	24	3	6	*p* < 0.05 (Corrected)	10
Yu et al. (2021)	Siemens (3.0T)	12-channel phased-array	2000	30	4	6	*p* < 0.05 (Corrected)	2
Zhao et al. (2019)	Siemens (3.0T)	Standard head coil	2000	30	4	4	*p* < 0.01 (Corrected)	10
BrainGluSchi	Siemens (3.0T)	12-channel	2000	29	3.5	8	NA	NA
COBRE	Siemens (3.0T)	12-channel	2000	29	3.5	8	NA	NA
NMorphCH	Siemens (3.0T)	NA	2500/2200	20/27	3/4	8	NA	NA

Note: ^a^The study consisted of two subgroups, and we treated it as two separate datasets.

Abbreviations: *FWHM* full width at half-maximum; *MRI* magnetic resonance imaging; *NA* not available; *ST* slice thickness; *T* Tesla; *TE* echo time; *TR* repetition time.

### ReHo comparisons in three fMRI datasets

In the BrainGluSchi dataset, no finding was reported after FWE-TFCE correction (Fig. S2A). In the COBRE dataset, compared with healthy controls, patients with schizophrenia showed decreased ReHo in the bilateral postcentral/precentral gyrus, left thalamus, bilateral calcarine fissure, right cuneus, right superior anterior cingulate cortex, right middle temporal gyrus, and right Rolandic operculum, while increased ReHo were found in the bilateral medial superior frontal gyrus, bilateral supplementary motor area, left calcarine fissure, and right middle frontal gyrus (Fig. S2B and Table S3). In the NMorphCH dataset, we only found decreased ReHo in the right postcentral/precentral gyrus (Fig. S2C and Table S4). We also applied a liberal threshold (*p* < 0.001, voxel-level uncorrected); the distribution of significant regions in the three datasets was similar, mainly concentrated in the postcentral/precentral gyrus, calcarine fissure, cuneus, medial frontal cortex, thalamus, supplementary motor area, anterior cingulate cortex, middle/superior temporal gyrus, and middle occipital gyrus (Fig. S3).

### ReHo changes in meta-analysis

As shown in Fig. 2 and Table 3, compared with healthy controls, patients with schizophrenia exhibited decreased ReHo in the bilateral postcentral gyrus, right precentral gyrus, and right middle occipital gyrus, while increased ReHo in the bilateral medial superior frontal gyrus.

**Table 3:** ReHo changes in patients with schizophrenia in the meta-analysis.

Brain regions	SDM-Z	*p* value	Peak MNI coordinates			Cluster size (voxels)	Heterogeneity test		Egger’s test
			*x*	*y*	*z*		*Q* (*p* value)	*I*^2^ (%)	*p* value
Schizophrenia < healthy control									
Right postcentral/precentral gyrus	−7.667	~0	48	−16	42	1848	8.578 (0.995)	0.695	0.641
Left postcentral gyrus	−6.398	0.006	−56	−18	38	91	8.819 (0.994)	0.186	0.846
Right middle occipital gyrus	−6.377	0.007	46	−72	6	81	6.763 (0.999)	0.727	0.974
Schizophrenia > healthy control									
Bilateral medial superior frontal gyrus	5.690	0.001	8	36	52	833	8.975 (0.993)	1.100	0.843

Abbreviations: *MNI* Montreal Neurological Institute; *Q* Cochran’s *Q* statistic; *ReHo* regional homogeneity; *SDM* seed-based *d* mapping.

### Sensitivity analyses

**Fig. 3:**
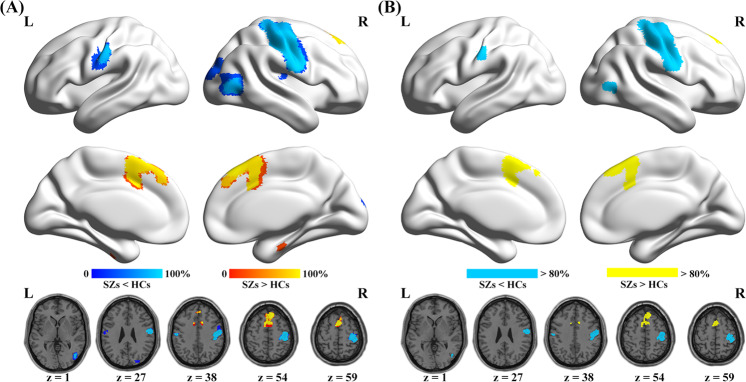
Results of the jackknife sensitivity analysis. **A** Voxel-wise probability map of significant clusters in jackknife sensitivity analysis, and the value in each voxel represents the probability of occurrence in all iterations; **B** the voxels with probability >80% in (**A**) were retained. Abbreviations: HCs healthy controls; L left; R right; SZs patients with schizophrenia.

**Fig. 4:**
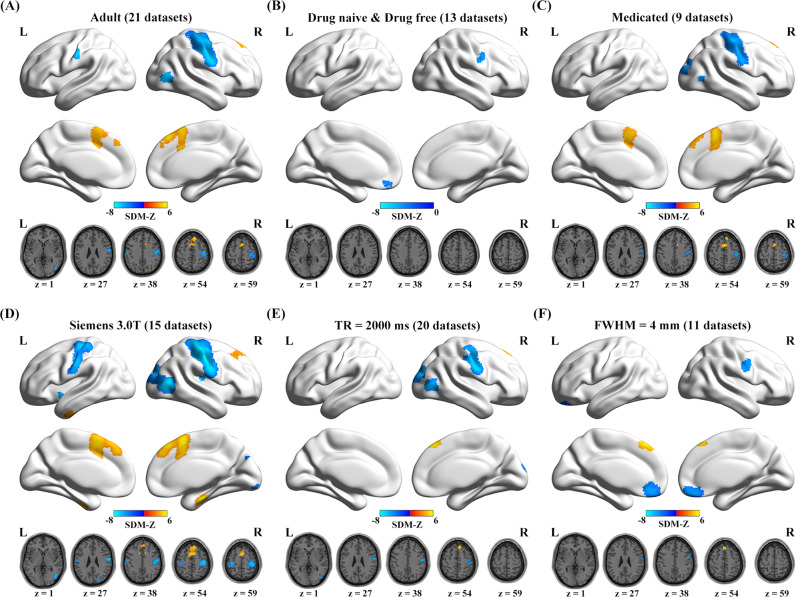
Results of the subgroup meta-analysis. ReHo changes in six specific subgroups: **A** adult subjects, **B** drug-naive/free patients, **C** medicated patients, **D** Siemens 3.0T MRI scanner, **E** TR = 2000 ms, **F** FWHM = 4 mm. The results were thresholded with FWE-TFCE correction *p* < 0.05. Abbreviations: FWE family-wise error; FWHM full width at half-maximum; L left; MRI magnetic resonance imaging; R right; ReHo regional homogeneity; SDM seed-based *d* mapping; T Tesla; TFCE threshold-free cluster enhancement; TR repetition time.

The probability map showed that all significant clusters were largely replicable in more than 80% iterations in the jackknife sensitivity analysis (Fig. 3A, B). In the subgroup analysis, after FWE-TFCE correction, findings in the adult subgroup (21 datasets, Fig. 4A and Table S5) and the Siemens 3.0T subgroup (15 datasets, Fig. 4D and Table S8) were largely consistent with the pooled meta-analysis. The drug-naive/free patient subgroup (13 datasets, Fig. 4B and Table S6) showed decreased ReHo in the left medial frontal cortex and right postcentral/precentral gyrus. The medicated patient subgroup (9 datasets, Fig. 4C and Table S7) showed decreased ReHo in the right postcentral/precentral gyrus, right superior occipital gyrus extending to right middle occipital gyrus, and right middle temporal gyrus, while increased ReHo in the bilateral medial superior frontal gyrus. In the subgroup of studies using TR = 2000 ms (20 datasets, Fig. 4E and Table S9), ReHo was found to be significantly increased in the right medial superior frontal gyrus, and decreased in the right postcentral/precentral gyrus, right superior occipital gyrus, and right middle occipital gyrus extending to right middle temporal gyrus. In the subgroup of studies that used a smooth kernel size of 4 mm (11 datasets, Fig. 4F and Table S10), schizophrenia group showed increased ReHo in the bilateral medial superior frontal gyrus, and decreased ReHo in the right postcentral/precentral gyrus and bilateral medial frontal cortex. Considering that fewer studies included in subgroup meta-analysis may lead to low statistical power, the results under a liberal threshold (*p* < 0.001, voxel-level uncorrected) were also shown in Fig. S4, which suggested that the main results were largely unchanged in different subgroups. Furthermore, heterogeneity tests revealed that none of the regions with altered ReHo identified in the pooled meta-analysis exhibited significant between-study heterogeneity (Table 3, all *ps* > 0.99 and *I*^2^s < 2%); Egger’s tests indicated that there was no significant publication bias in any region (Table 3 and Fig. S5, all *ps* > 0.6).

### Meta-regression analysis

The ReHo alterations in the right postcentral/precentral gyrus had a negative correlation with illness duration in patients with schizophrenia (*p* = 0.0218), that is, patients with longer illness duration had more ReHo reduction in this region. Additionally, the PANSS scores, mean age, and female-to-male ratio were not found to be correlated with ReHo changes, at least linearly.

## Discussion

In the current study, we performed an updated and extended meta-analysis with 815 schizophrenia patients and 790 healthy controls to identify consistent and reliable abnormalities of local FC. We mainly found increased local FC in the bilateral medial superior frontal gyrus, while a decrease in the bilateral postcentral gyrus, right precentral gyrus, and right middle occipital gyrus. A series of sensitivity analyses including jackknife, subgroup, and heterogeneity analyses demonstrated that these findings were highly reliable. Furthermore, meta-regression analysis revealed that illness duration was negatively correlated with local FC changes in the right precentral/postcentral gyrus.

Significantly decreased local connectivity was observed in the bilateral postcentral gyrus and right precentral gyrus, that is, the sensorimotor cortex. The precentral gyrus, comprising the primary motor cortex, is an important structure involved in the control of voluntary motor movement^54^. It has also been confirmed that the precentral gyrus plays a key part in the processing of various motor-related cognitive functions^55^. The postcentral gyrus contains the primary somatosensory cortex, and is responsible for proprioception. It perceives various somatic sensory stimuli from specific areas of the body, including tactile sensation, pressure, temperature, and pain^56^. In a word, reduced activation of the sensorimotor cortex in patients with schizophrenia is related to the dysfunction in sensory integration, motor regulation, and cognitive process. Evidence from previous studies is also in support of our findings^57,58^. An emerging role of the somatosensory cortex is emotion regulation^59^, and we could infer that it is relevant to negative symptoms of so-called emotional flatness (e.g., anhedonia, avolition, and lack of expressiveness) in schizophrenia patients. In addition, according to the results of our meta-regression analyses, longer course of disease had a more significant moderating influence on ReHo changes in the right precentral/postcentral gyrus, in other words, patients with chronic schizophrenia displayed a more significant reduction in ReHo values. Hence, we can draw the conclusion that local connectivity impairments in the sensorimotor cortex become more extensive as the disease progresses.

Located in the occipital lobe, the right middle occipital gyrus is a core part of the visual cortex, and is mainly responsible for the reception, segmentation, and integration of visual information^60^. The dysfunction of this region is therefore associated with visual perception disturbances among schizophrenia patients, such as visional hallucinations and distortions^61,62^. We found that ReHo values were significantly lower in the visual cortex as well, which is consistent with earlier findings of visual processing deficits and higher-order visual cognition impairments in schizophrenia^19^. In accordance with former studies, patients with schizophrenia experienced aberrant early-stage visual processing^63,64^. In the present study, we included patients both with first-episode and chronic schizophrenia with a mean illness duration ranging from 2 to 204 months, which indicated that the functional abnormalities in the visual cortex may persist throughout the course of the disease.

We found higher local connectivity in the bilateral medial superior frontal gyrus compared with healthy controls. These regions are located in the medial prefrontal cortex (MPFC), a major part of the default mode network (DMN). The DMN is predominantly activated during the resting state in order to enable higher levels of cognition and attention control, especially in working memory^65^. Thereinto, the MPFC plays crucial roles in the regulation of emotion, cognitive process, and behavior management^66^. Lesions of this area lead to the impairment of these functions, and have been implicated in a range of neurological and psychiatric disorders including schizophrenia^67^. In our research, significantly increased ReHo in the MPFC was identified, which represented deficits of these associated functions in schizophrenia.

As the subgroup meta-analyses demonstrated, after FWE-TFCE correction, no entirely consistent findings were reported in comparison with our main results. However, when an uncorrected threshold was applied, the results kept roughly the same (Fig. S4). This may be ascribed to the fact that a small number of studies (and therefore small sample sizes) have relatively low statistical power^16,17^. Consequently, under a strict significance level, fewer brain regions with significantly altered ReHo were identified. Besides, patients in the drug-naive/free group usually had a shorter illness duration and thus less brain damage, whereas the opposite was true in the medicated patient group. This may partially explain why we observed inconsistent results in the two subgroups, and medicated patients had more areas with significant ReHo alterations. In the meta-regression analysis, we found that mean age and female-to-male ratio were not associated with changes in ReHo, which suggests that the main results in our study were not influenced by the two main demographic characteristics. We also examined the association between PANSS, a medical scale used for rating the symptoms of schizophrenia^68^, and ReHo changes. We found no correlation between PANSS scores and ReHo alterations, and the reason may be twofold: on one hand, the sample size may limit the statistical power to detect significant correlations; on the other hand, the non-significant result suggested that ReHo abnormality is a stable trait in schizophrenia, independent of symptom severity.

Raw statistical maps of ReHo comparison in three independent datasets of schizophrenia were used in the meta-analysis, while there are inconsistencies among them. After FWE-TFCE correction, multiple regions with significantly altered ReHo were identified in the COBRE dataset, only a decrease in ReHo was found in the right postcentral/precentral gyrus in the NMorphCH dataset, and the BrainGluSchi dataset reported null findings. However, under the threshold of voxel-level uncorrected *p* < 0.001, we found similar patterns of impairments in schizophrenia in the three datasets. This indicates that it is difficult to obtain reliable results from a single study, and a meta-analysis is needed to unify findings of previous studies to reach a consistent conclusion.

There are several limitations in our study that need to be mentioned. First, only three raw statistical maps were included in this study. Although challenging in practice, multicenter data sharing to boost the sample size (and therefore statistical power) is highly needed, which contributes to more accurate results. Second, although subgroup analyses were conducted to explore the effect of variations in scanner, TR, and smooth kernel, there are still considerable variations in other aspects of data acquisition and processing, such as slice thickness and multiple correction method. Unfortunately, the effects of these variables cannot be examined due to the insufficient number of studies. Third, the relatively small sample size in most of the included studies might bias the results, which should be replicated in future research including studies with larger sample sizes. Fourth, most of the studies only recruited Chinese participants, which may limit the generalizability of the current findings to other populations. Finally, we chose ReHo in this study as it is a reliable and widely used metric to measure local FC in previous studies, but others including short-range functional connectivity density/strength (FCD/FCS) are also valid indicators of local FC^69,70^. Future studies should test the generalizability of our findings using other local FC indicators.

## Conclusion

In summary, our results provided evidence that schizophrenia is associated with abnormalities of local FC, particularly in the DMN, sensorimotor cortex, and visual cortex. Furthermore, a significant negative correlation was found between the altered local FC and illness duration in the right precentral/postcentral gyrus. These findings shed light on the pathophysiological mechanisms underlying schizophrenia, and future studies are needed to test whether the local FC abnormalities can serve as neuroimaging biomarkers in this disorder.

## Supplementary information

Supplementary Tables

Figure S1

Figure S2

Figure S3

Figure S4

Figure S5

Supplementary Figure Legends

## Data Availability

This study included three public resting-state fMRI datasets of schizophrenia (the BrainGluSchi, COBRE, and NMorphCH datasets), and they are available on the SchizConnect website (http://schizconnect.org/). Besides, all input datasets and result files for the current meta-analysis are publicly available at figshare (10.6084/m9.figshare.20235909.v3).
