# Supplementary material for: Disrupted local functional connectivity in schizophrenia: An updated and extended meta-analysis
Source: Schizophrenia (Heidelb). 2022 Nov 8;8(1):93. doi: 10.1038/s41537-022-00311-2 (PMC9643538; doi:10.1038/s41537-022-00311-2)
Supplement: Supplementary file 1 — Supplementary Tables [file 41537_2022_311_MOESM1_ESM.docx]

**Supplementary Tables**

Table S1. 10-point checklist of quality assessment.

| **Category 1: Participants** |
| --- |
| 1. Patients were evaluated prospectively, specific diagnostic criteria were applied, and demographic data were reported.  2. Comparison participants were evaluated prospectively; psychiatric and medical illnesses were excluded.  3. Important variables (e.g., age, sex, illness duration, onset, medication status, and handedness) were checked either by stratification or statistically.  4. Sample size per group > 10. |
| **Category 2: Methods for image acquisition and analysis** |
| 5. Whole brain analysis was automated without a priori regional selection.  6. Coordinates reported in a standard space.  7. The imaging technique used was clearly described so that it could be reproduced.  8. Measurements were clearly described so that they could be reproduced. |
| **Category 3: Results and conclusions** |
| 9. Statistical parameters for significant and important non-significant differences were provided.  10. Conclusions were consistent with the results obtained and the limitations were discussed. |

Note: The checklist is based on previous meta-analyses^1,2^.

Table S2. The detailed quality assessment scores for each study.

| Studies | 1 | 2 | 3 | 4 | 5 | 6 | 7 | 8 | 9 | 10 | Total |
| --- | --- | --- | --- | --- | --- | --- | --- | --- | --- | --- | --- |
| Bai et al. (2016) | 1 | 0.5 | 1 | 1 | 1 | 1 | 1 | 1 | 1 | 1 | 9.5 |
| Cui et al. (2016) | 1 | 1 | 1 | 1 | 1 | 1 | 1 | 1 | 1 | 1 | 10 |
| Gao et al. (2015) | 1 | 1 | 1 | 1 | 1 | 1 | 1 | 1 | 1 | 1 | 10 |
| Gao et al. (2018) | 1 | 1 | 1 | 1 | 1 | 1 | 1 | 1 | 1 | 1 | 10 |
| Gao et al. (2020) | 1 | 1 | 1 | 1 | 1 | 1 | 1 | 1 | 1 | 1 | 10 |
| Gou et al. (2018) | 1 | 1 | 1 | 1 | 1 | 1 | 1 | 1 | 1 | 1 | 10 |
| Hu et al. (2016) | 1 | 1 | 1 | 1 | 1 | 1 | 1 | 1 | 1 | 1 | 10 |
| Jin et al. (2021) | 0.5 | 1 | 0.5 | 1 | 1 | 1 | 1 | 1 | 1 | 1 | 9 |
| Liu et al. (2006) | 0.5 | 1 | 1 | 1 | 1 | 1 | 1 | 1 | 1 | 0.5 | 9 |
| Liu et al. (2016) | 1 | 0.5 | 1 | 1 | 1 | 1 | 1 | 1 | 1 | 1 | 9.5 |
| Lyu et al. (2021) | 1 | 1 | 1 | 1 | 1 | 1 | 1 | 1 | 1 | 1 | 10 |
| Shan et al. (2021) | 1 | 1 | 1 | 1 | 1 | 1 | 1 | 1 | 1 | 1 | 10 |
| Wang et al. (2017) | 1 | 0.5 | 1 | 1 | 1 | 1 | 1 | 1 | 1 | 0.5 | 9 |
| Yan et al. (2020) | 1 | 1 | 1 | 1 | 1 | 1 | 1 | 1 | 1 | 1 | 10 |
| Yang et al. (2021) | 1 | 1 | 1 | 1 | 1 | 1 | 1 | 1 | 1 | 1 | 10 |
| Yu et al. (2013) | 1 | 1 | 1 | 1 | 1 | 1 | 1 | 1 | 1 | 1 | 10 |
| Yu et al. (2021) | 1 | 1 | 1 | 1 | 1 | 1 | 1 | 1 | 1 | 1 | 10 |
| Zhao et al. (2019) | 1 | 1 | 1 | 1 | 1 | 1 | 1 | 1 | 1 | 0.5 | 9.5 |

Table S3. ReHo changes in patients with schizophrenia in the COBRE dataset.

| Brain regions | Peak MNI coordinates | | | *t* value | Cluster size  (voxels) |
| --- | --- | --- | --- | --- | --- |
|  | *x* | *y* | *z* |  |  |
| **Schizophrenia < healthy control** |  |  |  |  |  |
| Left postcentral/precentral gyrus/thalamus | -9 | -12 | 3 | -6.833 | 1770 |
| Right postcentral/precentral gyrus | 45 | -24 | 51 | -5.524 | 697 |
| Bilateral calcarine fissure/right cuneus | -9 | -90 | 0 | -4.410 | 603 |
| Right superior anterior cingulate cortex | 3 | 27 | 12 | -5.767 | 52 |
| Right middle temporal gyrus | 45 | -60 | 3 | -5.088 | 30 |
| Right Rolandic operculum | 39 | -21 | 18 | -3.203 | 3 |
| **Schizophrenia > healthy control** |  |  |  |  |  |
| Bilateral medial superior frontal gyrus | 0 | 42 | 45 | 4.463 | 201 |
| Bilateral supplementary motor area | 3 | 9 | 54 | 3.987 | 35 |
| Left calcarine fissure | -6 | -45 | 3 | 4.189 | 25 |
| Right middle frontal gyrus | 45 | 42 | 9 | 4.066 | 7 |

Abbreviations: MNI, Montreal Neurological Institute; ReHo, regional homogeneity.

Table S4. ReHo changes in patients with schizophrenia in the NMorphCH dataset.

| Brain regions | Peak MNI coordinates | | | *t* value | Cluster size  (voxels) |
| --- | --- | --- | --- | --- | --- |
|  | *x* | *y* | *z* |  |  |
| **Schizophrenia < healthy control** |  |  |  |  |  |
| Right postcentral/precentral gyrus | 48 | -24 | 51 | -5.087 | 288 |

Abbreviations: MNI, Montreal Neurological Institute; ReHo, regional homogeneity.

Table S5. ReHo changes in patients with schizophrenia in the adult subgroup.

| Brain regions | SDM-Z | *p* value | Peak MNI coordinates | | | Cluster size  (voxels) | Heterogeneity test | | Egger’s test |
| --- | --- | --- | --- | --- | --- | --- | --- | --- | --- |
|  |  |  | *x* | *y* | *z* |  | *Q* (*p* value) | *I*^2^ (%) | *p* value |
| **Schizophrenia < healthy control** |  |  |  |  |  |  |  |  |  |
| Right postcentral/precentral gyrus | -6.794 | ~0 | 48 | -16 | 42 | 1828 | 10.902 (0.949) | 1.619 | 0.695 |
| Right middle temporal/occipital gyrus | -6.164 | 0.008 | 46 | -72 | 6 | 88 | 3.985 (0.999) | 0.571 | 0.999 |
| Left postcentral gyrus | -6.114 | 0.012 | -56 | -18 | 38 | 49 | 8.273 (0.990) | 0.253 | 0.930 |
| **Schizophrenia > healthy control** |  |  |  |  |  |  |  |  |  |
| Bilateral supplementary motor area/medial superior frontal gyrus | 5.597 | 0.001 | 6 | 34 | 52 | 612 | 9.833 (0.971) | 0.346 | 0.681 |
| Left medial superior frontal gyrus | 4.051 | 0.021 | -6 | 36 | 42 | 14 | 11.351 (0.937) | 0.926 | 0.762 |
| Right medial superior frontal gyrus | 3.871 | 0.024 | 4 | 42 | 38 | 3 | 12.678 (0.891) | 5.133 | 0.935 |

Abbreviations: MNI, Montreal Neurological Institute; *Q*, Cochran’s *Q* statistic; ReHo, regional homogeneity; SDM, seed-based *d* mapping.

Table S6. ReHo changes in patients with schizophrenia in the drug-naive/free patient subgroup.

| Brain regions | SDM-Z | *p* value | Peak MNI coordinates | | | Cluster size  (voxels) | Heterogeneity test | | Egger’s test |
| --- | --- | --- | --- | --- | --- | --- | --- | --- | --- |
|  |  |  | *x* | *y* | *z* |  | *Q* (*p* value) | *I*^2^ (%) | *p* value |
| **Schizophrenia < healthy control** |  |  |  |  |  |  |  |  |  |
| Left medial frontal cortex | -4.595 | 0.012 | -8 | 42 | -18 | 65 | 4.040 (0.983) | 2.357 | 0.517 |
| Right postcentral/precentral gyrus | -4.682 | 0.013 | 58 | -4 | 32 | 40 | 5.641 (0.933) | 8.410 | 0.854 |

Abbreviations: MNI, Montreal Neurological Institute; *Q*, Cochran’s *Q* statistic; ReHo, regional homogeneity; SDM, seed-based *d* mapping.

Table S7. ReHo changes in patients with schizophrenia in the medicated patient subgroup.

| Brain regions | SDM-Z | *p* value | Peak MNI coordinates | | | Cluster size  (voxels) | Heterogeneity test | | Egger’s test |
| --- | --- | --- | --- | --- | --- | --- | --- | --- | --- |
|  |  |  | *x* | *y* | *z* |  | *Q* (*p* value) | *I*^2^ (%) | *p* value |
| **Schizophrenia < healthy control** |  |  |  |  |  |  |  |  |  |
| Right postcentral/precentral gyrus | -5.785 | 0.001 | 42 | -22 | 56 | 1121 | 3.233 (0.919) | 3.371 | 0.536 |
| Right superior/middle occipital gyrus | -5.072 | 0.007 | 20 | -94 | 14 | 114 | 2.563 (0.959) | 0.852 | 0.822 |
| Right middle temporal gyrus | -5.213 | 0.021 | 44 | -70 | 4 | 3 | 1.740 (0.988) | 1.639 | 0.983 |
| **Schizophrenia > healthy control** |  |  |  |  |  |  |  |  |  |
| Bilateral medial superior frontal gyrus | 5.542 | ~0 | 2 | 12 | 56 | 599 | 0.928 (0.999) | 1.422 | 0.794 |
| Right medial superior frontal gyrus | 4.615 | 0.011 | 8 | 36 | 54 | 64 | 1.235 (0.996) | 0.801 | 0.850 |
| Right medial superior frontal gyrus | 4.090 | 0.022 | 6 | 44 | 42 | 12 | 6.534 (0.588) | 12.716 | 0.942 |

Abbreviations: MNI, Montreal Neurological Institute; *Q*, Cochran’s *Q* statistic; ReHo, regional homogeneity; SDM, seed-based *d* mapping.

Table S8. ReHo changes in patients with schizophrenia in the subgroup of studies using Siemens 3.0T MRI scanner.

| Brain regions | SDM-Z | *p* value | Peak MNI coordinates | | | Cluster size  (voxels) | Heterogeneity test | | Egger’s test |
| --- | --- | --- | --- | --- | --- | --- | --- | --- | --- |
|  |  |  | *x* | *y* | *z* |  | *Q* (*p* value) | *I*^2^ (%) | *p* value |
| **Schizophrenia < healthy control** |  |  |  |  |  |  |  |  |  |
| Right postcentral/precentral gyrus | -7.440 | 0.001 | 42 | -20 | 54 | 2630 | 7.484 (0.914) | 8.632 | 0.831 |
| Right middle occipital/temporal gyrus | -6.762 | 0.001 | 44 | -70 | 6 | 902 | 6.928 (0.937) | 1.240 | 0.991 |
| Left postcentral/precentral gyrus | -6.186 | 0.001 | -36 | -24 | 62 | 887 | 4.735 (0.989) | 2.630 | 0.890 |
| Left cerebellum/Right lingual gyrus | -5.058 | 0.016 | 0 | -84 | -18 | 108 | 7.672 (0.906) | 0.545 | 0.941 |
| Left insula | -5.813 | 0.016 | -42 | 8 | -12 | 46 | 2.287 (0.999) | 0.222 | 0.788 |
| Right insula | -5.197 | 0.025 | 36 | -20 | 14 | 5 | 5.477 (0.978) | 0.190 | 0.980 |
| **Schizophrenia > healthy control** |  |  |  |  |  |  |  |  |  |
| Bilateral medial superior frontal gyrus | 6.055 | ~0 | 2 | 12 | 56 | 1658 | 5.405 (0.979) | 1.208 | 0.834 |
| Right parahippocampal gyrus | 5.636 | 0.005 | 24 | -16 | -24 | 255 | 2.348 (0.999) | 0.490 | 0.690 |
| Left inferior temporal gyrus/fusiform gyrus | 5.096 | 0.005 | -40 | -12 | -34 | 273 | 5.020 (0.986) | 0.023 | 0.980 |
| Right fusiform gyrus | 3.912 | 0.024 | 30 | -8 | -38 | 7 | 7.536 (0.912) | 0.387 | 0.987 |

Abbreviations: MNI, Montreal Neurological Institute; MRI, magnetic resonance imaging; *Q*, Cochran’s *Q* statistic; ReHo, regional homogeneity; SDM, seed-based *d* mapping; T, Tesla.

Table S9. ReHo changes in patients with schizophrenia in the subgroup of studies using TR = 2000 ms.

| Brain regions | SDM-Z | *p* value | Peak MNI coordinates | | | Cluster size  (voxels) | Heterogeneity test | | Egger’s test |
| --- | --- | --- | --- | --- | --- | --- | --- | --- | --- |
|  |  |  | *x* | *y* | *z* |  | *Q* (*p* value) | *I*^2^ (%) | *p* value |
| **Schizophrenia < healthy control** |  |  |  |  |  |  |  |  |  |
| Right postcentral/precentral gyrus | -6.542 | 0.001 | 50 | -14 | 40 | 589 | 9.171 (0.971) | 0.258 | 0.606 |
| Right superior/middle occipital gyrus | -5.635 | 0.002 | 26 | -86 | 24 | 335 | 8.797 (0.977) | 0.122 | 0.954 |
| Right middle occipital/temporal gyrus | -5.740 | 0.004 | 46 | -72 | 6 | 114 | 3.645 (0.999) | 0.664 | 0.899 |
| **Schizophrenia > healthy control** |  |  |  |  |  |  |  |  |  |
| Right medial superior frontal gyrus | 5.490 | 0.008 | 6 | 34 | 52 | 96 | 9.528 (0.964) | 0.810 | 0.653 |

Abbreviations: MNI, Montreal Neurological Institute; *Q*, Cochran’s *Q* statistic; ReHo, regional homogeneity; SDM, seed-based *d* mapping; TR, repetition time.

Table S10. ReHo changes in patients with schizophrenia in the subgroup of studies using a smooth kernel size of 4 mm.

| Brain regions | SDM-Z | *p* value | Peak MNI coordinates | | | Cluster size  (voxels) | Heterogeneity test | | Egger’s test |
| --- | --- | --- | --- | --- | --- | --- | --- | --- | --- |
|  |  |  | *x* | *y* | *z* |  | *Q* (*p* value) | *I*^2^ (%) | *p* value |
| **Schizophrenia < healthy control** |  |  |  |  |  |  |  |  |  |
| Bilateral medial frontal cortex | -5.134 | 0.001 | 0 | 40 | -18 | 701 | 1.792 (0.994) | 1.172 | 0.725 |
| Right postcentral/precentral gyrus | -4.826 | 0.011 | 58 | -6 | 30 | 119 | 4.611 (0.867) | 3.625 | 0.839 |
| **Schizophrenia > healthy control** |  |  |  |  |  |  |  |  |  |
| Bilateral medial superior frontal gyrus | 5.951 | 0.004 | 0 | 32 | 52 | 117 | 3.269 (0.953) | 1.251 | 0.543 |

Abbreviations: MNI, Montreal Neurological Institute; *Q*, Cochran’s *Q* statistic; ReHo, regional homogeneity; SDM, seed-based *d* mapping.

**References**

1. Wang, T., et al. Altered resting-state functional activity in posttraumatic stress disorder: A quantitative meta-analysis. *Sci Rep.* 2016; 6:27131.

2. Shepherd, A. M., et al. Systematic meta-review and quality assessment of the structural brain alterations in schizophrenia. *Neurosci Biobehav Rev.* 2012; 36(4):1342-1356.
