# Supplementary figures and images for: Disrupted local functional connectivity in schizophrenia: An updated and extended meta-analysis
Source: Schizophrenia (Heidelb). 2022 Nov 8;8(1):93. doi: 10.1038/s41537-022-00311-2 (PMC9643538; doi:10.1038/s41537-022-00311-2)

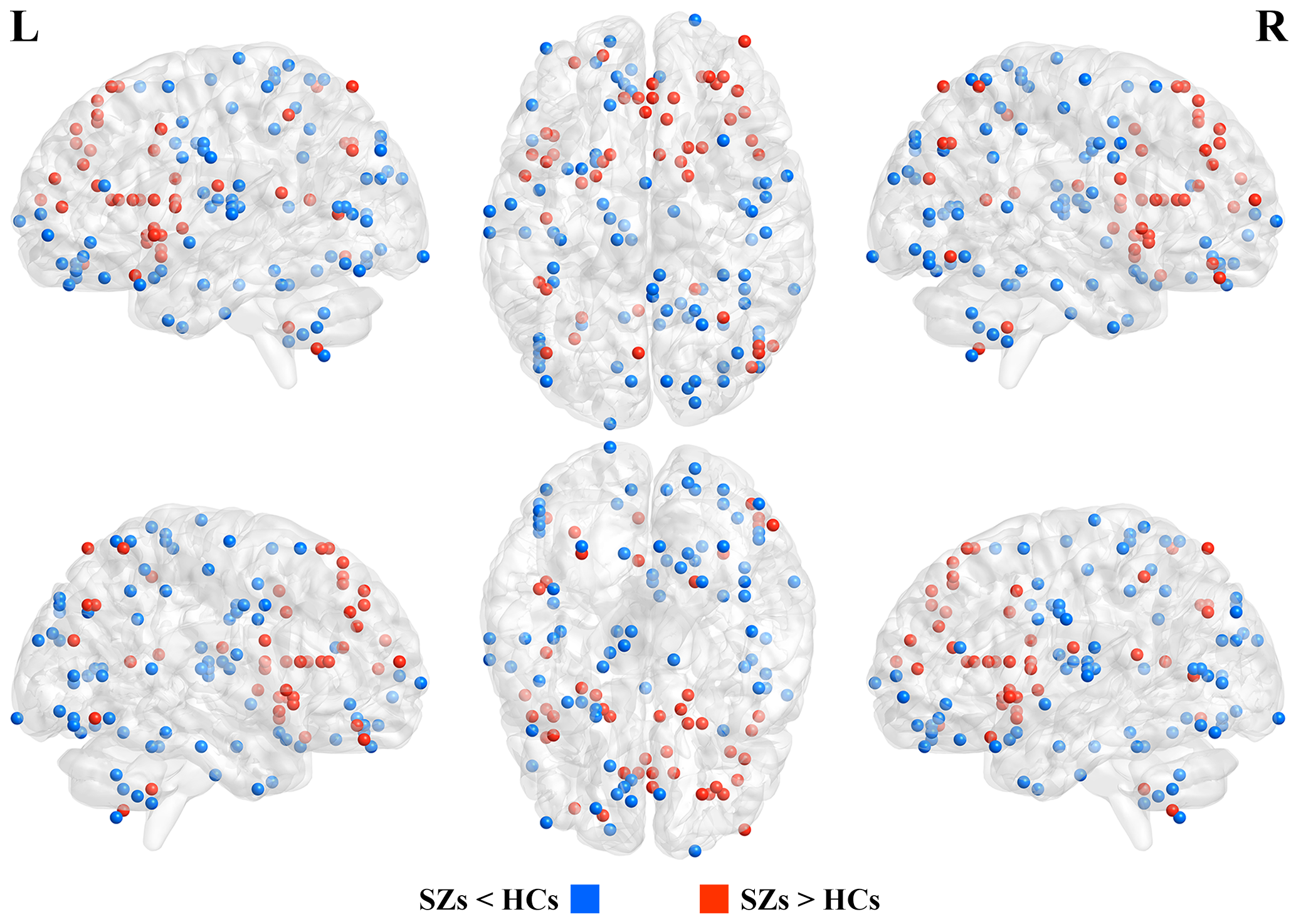

Supplement: Supplementary file 2 — Figure S1 [file 41537_2022_311_MOESM2_ESM.tif]

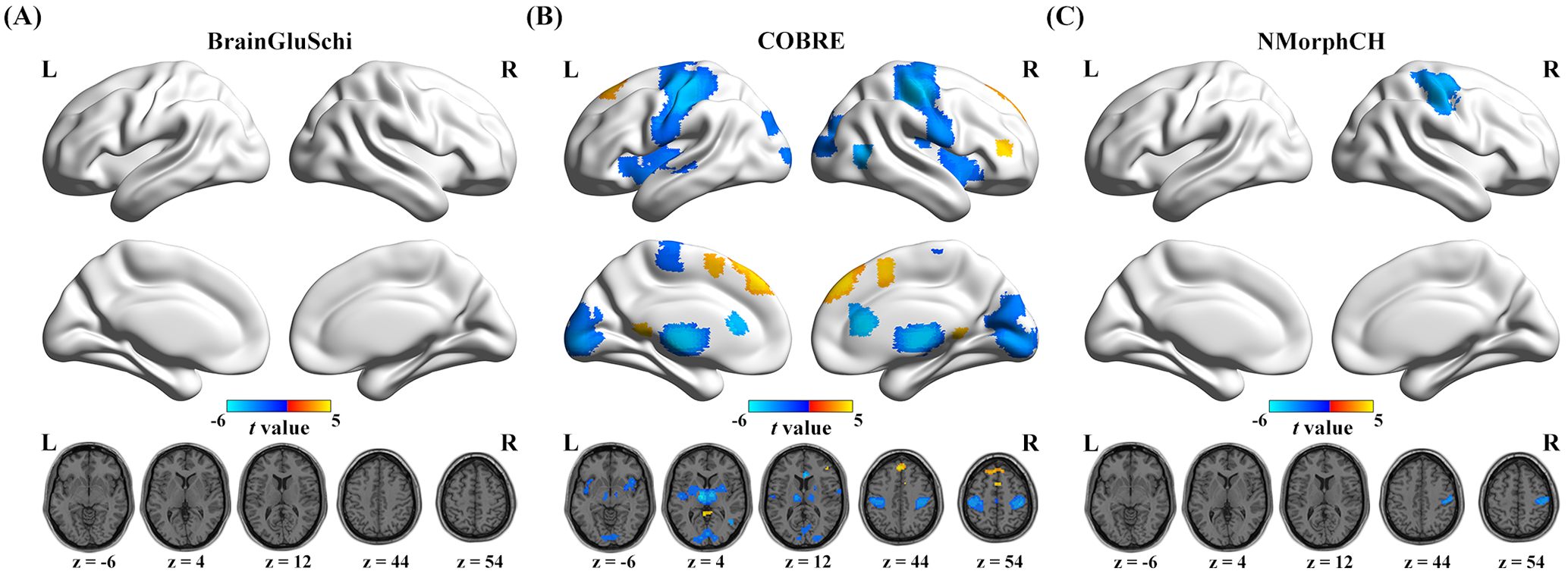

Supplement: Supplementary file 3 — Figure S2 [file 41537_2022_311_MOESM3_ESM.tif]

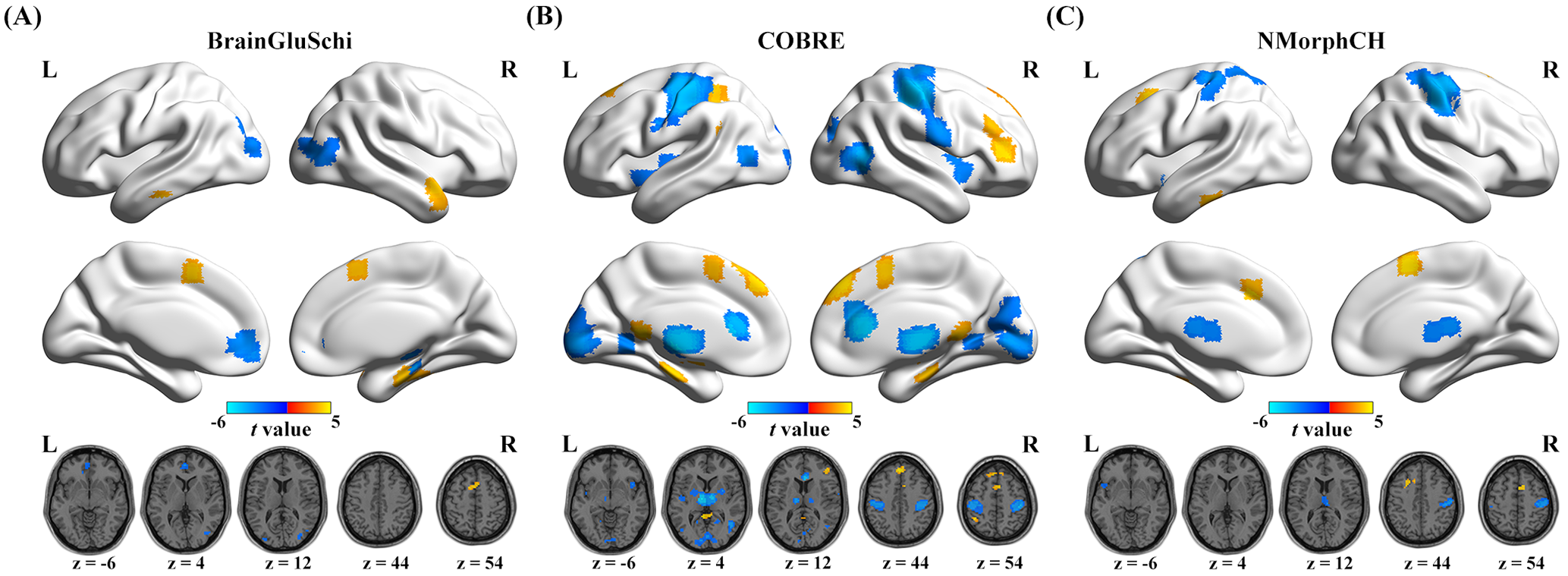

Supplement: Supplementary file 4 — Figure S3 [file 41537_2022_311_MOESM4_ESM.tif]

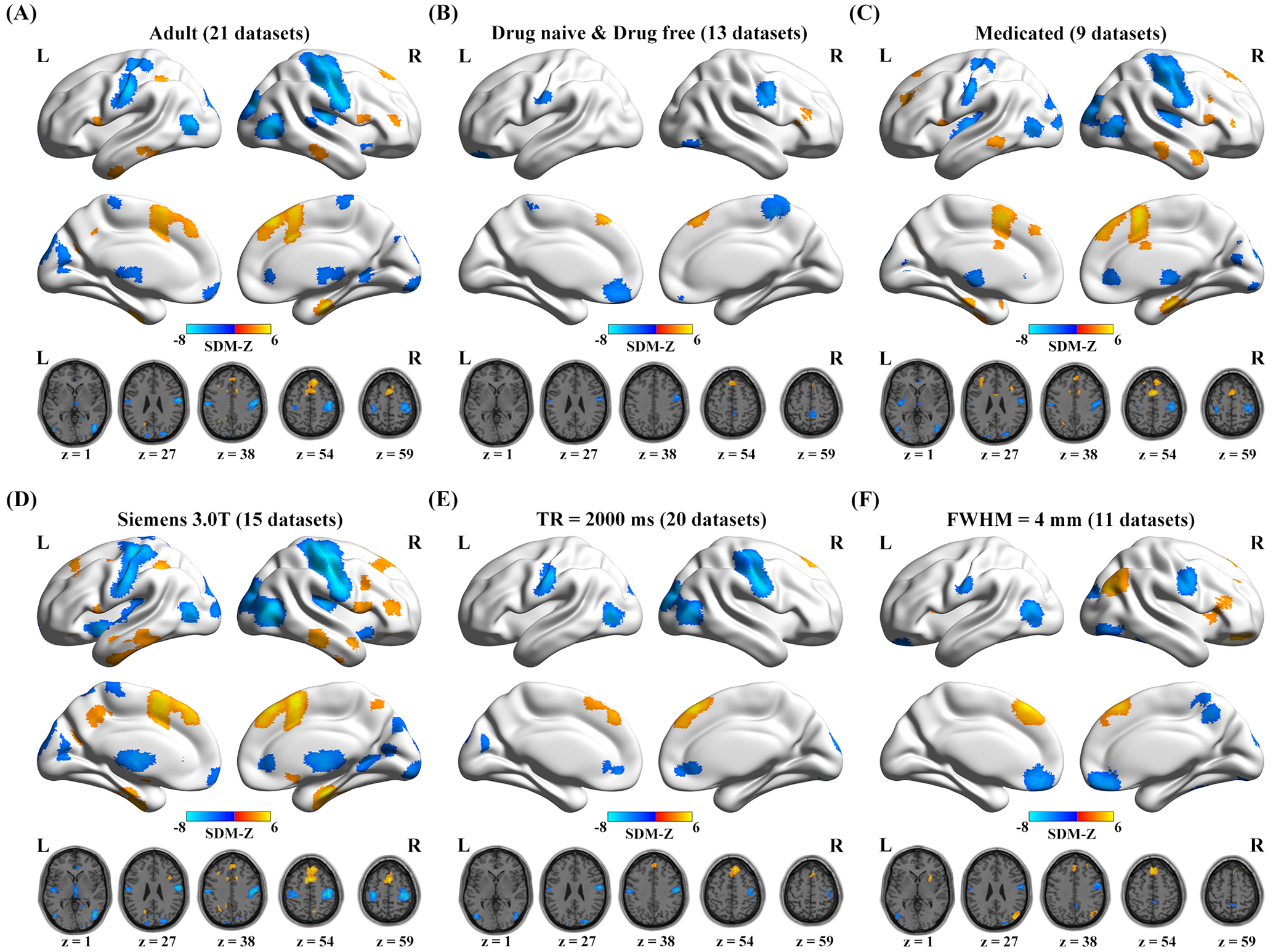

Supplement: Supplementary file 5 — Figure S4 [file 41537_2022_311_MOESM5_ESM.tif]

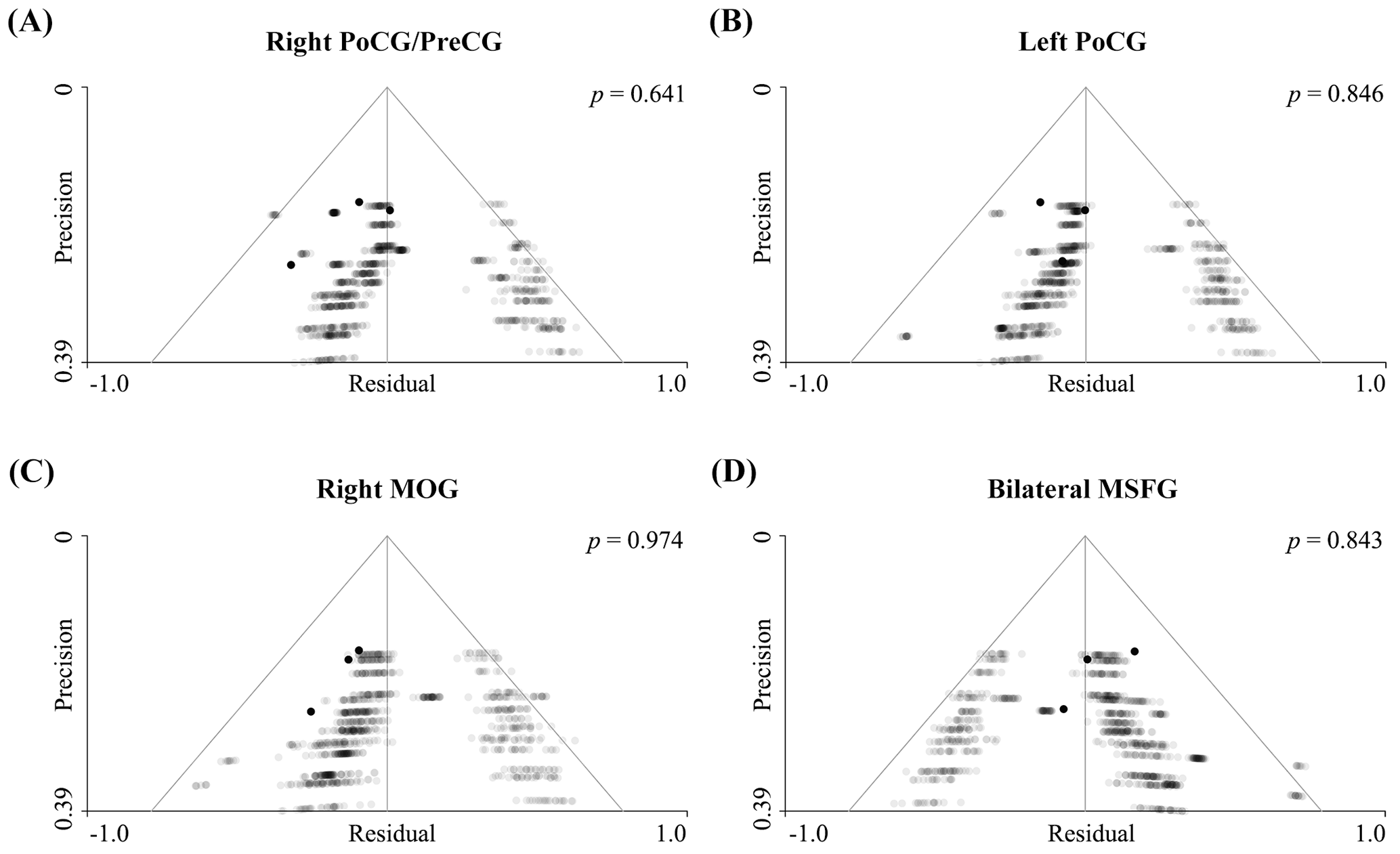

Supplement: Supplementary file 6 — Figure S5 [file 41537_2022_311_MOESM6_ESM.tif]
