# Supplementary material for: Disrupted local functional connectivity in schizophrenia: An updated and extended meta-analysis
Source: Schizophrenia (Heidelb). 2022 Nov 8;8(1):93. doi: 10.1038/s41537-022-00311-2 (PMC9643538; doi:10.1038/s41537-022-00311-2)
Supplement: Supplementary file 7 — Supplementary Figure Legends [file 41537_2022_311_MOESM7_ESM.docx]

**Supplementary figure legends**

Fig. S1. Plots of all coordinates included in the meta-analysis with red and blue spheres indicating significantly increased and decreased ReHo in original studies, respectively.

Abbreviations: HCs, healthy controls; L, left; R, right; ReHo, regional homogeneity; SZs, patients with schizophrenia.

Fig. S2. ReHo changes in patients with schizophrenia in three fMRI datasets: (A) the BrainGluSchi dataset, (B) the COBRE dataset, (C) the NMorphCH dataset. The results were thresholded with FWE-TFCE correction *p* < 0.05.

Abbreviations: FWE, family-wise error; L, left; R, right; ReHo, regional homogeneity; TFCE, threshold-free cluster enhancement.

Fig. S3. ReHo changes in patients with schizophrenia in three fMRI datasets: (A) the BrainGluSchi dataset, (B) the COBRE dataset, (C) the NMorphCH dataset. The results were thresholded with voxel-level uncorrected *p* < 0.001.

Abbreviations: L, left; R, right; ReHo, regional homogeneity.

Fig. S4. Results of the subgroup meta-analysis. ReHo changes in six specific subgroups: (A) adult subjects, (B) drug-naive/free subjects, (C) medicated subjects, (D) Siemens 3.0T MRI scanner, (E) TR = 2000 ms, (F) FWHM = 4 mm. The results were thresholded with voxel-level uncorrected *p* < 0.001.

Abbreviations: FWHM, full width at half-maximum; L, left; MRI, magnetic resonance imaging; R, right; ReHo, regional homogeneity; SDM, seed-based *d* mapping; T, Tesla; TR, repetition time.

Fig. S5. Funnel plots of regions with significant ReHo changes in schizophrenia. Each dataset is marked as a dot.

Abbreviations: MOG, middle occipital gyrus; MSFG, medial superior frontal gyrus; PoCG, postcentral gyrus; PreCG, precentral gyrus; ReHo, regional homogeneity.
